# The Associations Between Happiness Motives and Well-Being in China: The Mediating Role of Psychological Need Satisfaction and Frustration

**DOI:** 10.3389/fpsyg.2020.02198

**Published:** 2020-09-03

**Authors:** Li Lin, Hoi-Wing Chan

**Affiliations:** ^1^Department of Applied Social Sciences, The Hong Kong Polytechnic University, Kowloon, Hong Kong; ^2^Department of Marketing, The Chinese University of Hong Kong, Shatin, Hong Kong

**Keywords:** hedonic motives, eudaimonic motives, psychological need satisfaction, psychological need frustration, well-being

## Abstract

Happiness can be pursued based on hedonic motives (i.e., seeking pleasure and comfort) and/or eudaimonic motives (i.e., seeking to develop and make the best use of the self). Substantial studies have found that hedonic and eudaimonic motives relate to well-being outcomes in different ways. However, these findings were predominantly based on Western samples, while study about the relationship between happiness motives and well-being outcomes in Eastern cultures is scanty. Furthermore, little is known about the mechanisms that underlie these associations. To address these gaps, we conducted two studies based on Chinese college students. In study 1 (*N* = 301), structural equation modeling demonstrated that eudaimonic motives were positively associated with life satisfaction and meaning in life, but hedonic motives were not significantly associated with either indicator of well-being. In study 2 (*N* = 526), structural equation modeling showed that (1) hedonic motives had an indirect effect on life satisfaction through need frustration and (2) eudaimonic motives had indirect effects on life satisfaction and meaning in life through need satisfaction and need frustration. These findings highlight the important roles that the satisfaction and the frustration of basic psychological needs play in translating happiness motives into well-being outcomes.

## Introduction

Happiness, an objective that most people long to obtain, can be understood *via* two distinct yet complementary perspectives: a hedonic perspective and a eudaimonic perspective (Ryan and Deci, 2001). While the former regards the presence of pleasure and the absence of pain as central to happiness, the latter considers self-actualization, acquisition of meaning, and virtuous functioning as essential to happiness (Huta and Waterman, 2014). This conceptual distinction leads to a differentiation between hedonic well-being [or subjective well-being (SWB)] and eudaimonic well-being (EWB). Initially, Diener (1984) posited that happiness is manifested in affective responses and cognitive evaluations of life conditions, which was termed SWB. SWB is typically indexed by life satisfaction and a preponderance of positive over negative emotions (Diener et al., 1999). However, Ryff (1989) argued that SWB could not reflect the eudaimonic side of well-being and proposed an alternative perspective to understand well-being, which is coined psychological well-being. In her conception, central to well-being is positive functioning, and it encompasses six distinct dimensions of well-being (i.e., autonomy, environmental mastery, personal growth, positive relations with others, purpose in life, and self-acceptance) (Ryff and Keyes, 1995). In line with this eudaimonic conception, there are plentiful different perspectives about well-being. EWB is marked by, for example, the satisfaction of basic psychological needs (Ryan and Deci, 2000), personal expressiveness (Waterman et al., 2008), experience of meaning (Steger et al., 2006), and psychosocial flourishing (Diener et al., 2010). Previous research has pointed to the distinction of SWB and EWB in their structure (e.g., Joshanloo, 2016) as well as their associations with subjective experience (e.g., Vittersø and Søholt, 2011) and dispositional factors (e.g., value orientations; Joshanloo and Ghaedi, 2009).

SWB and EWB reflect one’s subjective feelings and experiences of happiness, which result from a prolonged engagement in certain ways of living that can be readily chosen and altered (Huta, 2016). Investigating different ways of living can help us understand how we can obtain SWB/EWB. Ways of living can be manifested in one’s motives behind daily activities, and these motives can be categorized as hedonic and eudaimonic ones (Huta and Ryan, 2010). In the current research, we seek to understand how hedonic and eudaimonic motives relate to SWB and EWB. First, study 1 attempted to extend previous studies from Western cultures (Huta, 2016) to Chinese culture and investigated the associations between hedonic and eudaimonic motives and SWB/EWB. Differently from Western cultures, Chinese people tend to downplay positive emotions and personal enjoyment (Joshanloo, 2014; Lu and Gilmour, 2004). It is unclear whether the findings based on Western samples can be generalized to Chinese ones. Second, as it is still unknown how hedonic and eudaimonic motives translate into feelings and experiences of well-being, study 2 attempted to unravel the psychological mechanisms that underlie these links. Based on self-determination theory (SDT; Ryan and Deci, 2017), particularly the basic psychological needs theory (BPNT; Ryan and Deci, 2000), we proposed that hedonic motives and eudaimonic motives contribute to SWB/EWB through the mediation of the satisfaction and the frustration of basic psychological needs for autonomy, relatedness, and competence. The current findings would advance our understanding of which way of living better contributes to SWB/EWB in Eastern collectivist cultures and to fill the gaps regarding why hedonic and eudaimonic motives relate or do not relate to SWB/EWB.

### Hedonic and Eudaimonic Motives and Well-Being

Huta and Ryan (2010) proposed hedonic and eudaimonic motives for activities (HEMA) as a fundamental concept for capturing different ways of living and explicating different pathways to happiness. These motives denote the intentions or aims that underlie daily activities. While hedonic motives concern boosting positive emotions, including seeking pleasure (i.e., positive emotions and psychological contentment) and comfort (i.e., relaxation and effortlessness), eudaimonic motives pertain to striving for optimization of a wide range of psychological functioning, including authenticity (i.e., acting in accord with the self), meaning (i.e., something greater than the self and of genuine importance), excellence (i.e., high standards and quality in morality and performance), and growth (i.e., acquisition of knowledge and skills and fulfillment of potentials) (Huta, 2016).

Studies have shown that hedonic and eudaimonic motives relate differently to a variety of SWB and EWB indicators. A summary of the previous findings about their correlations with well-being is presented in Table 1 [for other unpublished studies, see Huta (2016)]. For example, Huta and Ryan (2010) found that hedonic (vs. eudaimonic) motives had stronger associations with carefreeness, while eudaimonic (vs. hedonic) motives had stronger associations with the experience of meaning and elevating experience. In addition, they found that hedonic and eudaimonic motives were both positively linked with life satisfaction and vitality. They also conducted an intervention study in which the participants were required to increase activities with hedonic motives or those with eudaimonic motives. The results showed that increasing activities with hedonic motives led to an immediate improvement in more well-being indicators (i.e., more positive affect, carefreeness, vitality, life satisfaction, and less negative affect) than increasing those with eudaimonic motives (i.e., more meaning and less negative affect); however, increasing activities with eudaimonic motives demonstrated a greater long-term (i.e., 3 months) impact on more well-being indicators (i.e., more positive affect, carefreeness, vitality, elevating experience, and less negative affect) than increasing those with hedonic motives (i.e., more carefreeness and vitality and less negative affect). Beyond self-reported well-being, Kryza-Lacombe et al. (2019) found that eudaimonic motives, not hedonic motives, were related to higher academic achievement among university students in terms of grade point average. Overall, hedonic motives convey more instant benefits to well-being, particularly SWB, whereas eudaimonic motives demonstrate broader and longer-term benefits to well-being, including both SWB and EWB.

**TABLE 1:** Correlations between happiness motives and well-being indicators reported in the previous studies.

Author (year of publication)	Participants	Methodology	Correlations of hedonic motives and well-being (*r*)	Correlations of eudaimonic motives and well-being (*r*)
Huta and Ryan (2010)	College students	Study 1 and study 2: correlational. Study 3: experience-sampling and correlational. Study 4: intervention study (experiment).	Positive affect [studies 1, 2, and 4 (+++), study 3 (++)], negative affect [study 1 (−), study 4 (−−−), studies 2 and 3 (×)], carefreeness [studies 1 and 4 (+++), study 2 (+), study 3 (++)], meaning [study 1 (+++), studies 2 and 4 (++), study 3 (×)], elevation [study 1 (++), studies 2, 3, and 4 (×)], vitality [studies 1 and 4 (+++), study 2 (+), study 3 (++)], life satisfaction [study 1 (+++), study 3 (++), studies 2 and 4 (×)]	Positive affect [studies 1–4 (++)], negative affect [study 2 (−), studies 1, 3, and 4 (×)], carefreeness [study 1 (+), studies 2–4 (×)], meaning [studies 1, 2, and 4 (+++), study 3 (×)], elevation [studies 1–4 (+++)], vitality [studies 1, 2, and 4 (+++), study 3 (++)], life satisfaction [studies 1 and 2 (++), studies 3 and 4 (×)]
Huta et al. (2012)	College students	Cross-sectional and correlational.	Well-being of close others. Positive affect (+), negative affect (×), carefreeness (×), meaning (×), elevation (×), self-connectedness (×), vitality (×), and self-esteem (×)	Well-being of close others. Positive affect (++), meaning (++), elevation (++), self-connectedness (+), vitality (++), negative affect (×), carefreeness (×), and self-esteem (×)
Asano et al. (2018)	College students	Longitudinal	Hedonic pleasure: life satisfaction [study 1 (+++)], positive affect [study 1 (+++) and 2 (+)], personal growth [study 1 (++)], purpose in life [study 1 (+)], sense of meaning [study 2 (++)], negative affect (×), calm affect (×)] Hedonic comfort: life satisfaction [study 1 (+++)], positive affect [study 1 (+) and study 2 (×)], calm affect [study 1 (+) and study 2 (×)], personal growth [study 1 (+)], negative affect [studies 1 and 2 (×)], purpose in life (×), and sense of meaning (×)	Eudaimonic: life satisfaction [study 1 (+++)], positive affect [study 1 (+) and study 2 (×)], personal growth [study 1 (+++)], purpose in life [study 1 (+)], sense of meaning [study 2 (+++)], negative affect (×), calm affect (×)
Saunders et al. (2018)	Adults who completed a cardiac rehabilitation program for secondary prevention	Three-month prospective design	Hedonic (baseline): hedonic well-being baseline (×), hedonic well-being 3-month (+++), eudaimonic well-being [baseline and 3-month (+++)]. Hedonic (3-month follow-up): hedonic well-being [baseline and 3-month (+++)], eudaimonic well-being [baseline and 3-month (+++)]	Eudaimonic (baseline): eudaimonic well-being [baseline and 3-month (+++)]. Eudaimonic (3-month follow-up): hedonic well-being [3-month (+++)], eudaimonic well-being [baseline and 3-month (+++)]
Passmore et al. (2018)	College students	Cross-sectional and correlational	Affect balance [study 1 (+)], flourishing [study 1 (++)]	Affect balance [study 1 (+++)], flourishing [study 1 (+++)]
Kryza-Lacombe et al. (2019)	College students	Cross-sectional and correlational	Grade point average (×), depression (×), stress (×), anxiety (×)	GPA (++), depression (−−−), stress (−−), anxiety (×)
Braaten et al. (2019)	College students	Cross-sectional and correlational	Well-being experience derived from school: positive affect (+++), negative affect (×), school satisfaction (++), meaning (++), elevation (+++), self-connectedness (++), interest (+), vitality (+++)	Well-being experience derived from school: positive affect (+++), negative affect (−), school satisfaction (+++), meaning (+++), elevation (+++), self-connectedness (+++), interest (+++), vitality (+++)

**According to the guideline of Funder and Ozer (2019), effect sizes (r) of 0.1, 0.2, and 0.3 indicate small, medium, and large effects, respectively. The signs +, ++, and +++ indicate small (r = 0.10–0.20), medium (r = 0.20–0.30), and large effects (r > 0.30), respectively, for significant positive correlations. The signs −, −−, and −−− indicate small (r = −0.20 to −0.10), medium (r = −30 to −0.20), and large effects (r < −0.30), respectively, for significant negative correlations. Besides that, × indicates non-significant correlations*.*

These findings are in line with the substantial studies using the theoretical framework of happiness pursuits of Peterson et al. (2005)—orientations to happiness (e.g., Park et al., 2009; Yang et al., 2017). Peterson et al. (2005) identified three orientations to happiness—life of pleasure, life of meaning, and life of engagement to describe different ways of living. While life of pleasure describes a hedonic way of living (e.g., “For me, the good life is the pleasurable life”; “I go out of my way to feel euphoric”), life of meaning describes a eudaimonic one (e.g., “What I do matters to society”; “My life has a lasting meaning”). Differently from the HEMA framework that pertains to motives only, orientation to happiness is a broad concept that involves a constellation of beliefs, evaluation, preferences, and behaviors. This line of seminal work provides us a profile of individuals who live in hedonic and eudaimonic lives, respectively, and yields abundant evidence showing that the hedonic and the eudaimonic ways of living may contribute to well-being in a unique way. However, the mixture of components within one orientation hinders us from understanding what exactly (e.g., beliefs, preferences, or behavior?) leads to well-being. Focusing on the motives alone allows us to identify what exactly is involved in the pathway to well-being and how this component translates into well-being. Additionally, due to the single component, the HEMA framework also enables us to directly compare the effects of hedonic and eudaimonic ways of living and understand their relative contribution to well-being.

However, almost all the studies on happiness motives have been conducted in Western societies [see one exception from Japan (Asano et al., 2018) and one exception from China (Lai et al., 2020)]. Cross-cultural studies have pointed to the possibility of different understandings and attitudes toward hedonism between Western individualist societies and Eastern collectivist societies (Lu and Gilmour, 2004; Oishi and Gilbert, 2016). Hedonia is centered on personal enjoyment. Eastern individualist cultures often emphasize social obligations and collective interests and may thus view personal enjoyment in a less positive light. In contrast, personal enjoyment is deemed to be more desirable in Western individualist cultures as these cultures emphasize personal desires and self-worth (Oishi and Gilbert, 2016). Considering the inconsistency of hedonism with the collectivist culture, Joshanloo and Jarden (2016) argued that the positive relationship between hedonism and well-being would be weaker in collectivist (vs. individualist) cultures. In contrast, eudaimonic motives are aligned with self-improvement and social contributions, attributes that are valued in collectivist cultures (Joshanloo, 2014), and thus their associations with well-being would presumably be similar across individualist and collectivist cultures. Additionally, previous research has found that hedonic and eudaimonic well-being show different associations with dispositional factors (e.g., self-concept consistency; Church et al., 2014) in different countries, suggesting that the antecedents of hedonic and eudaimonic well-being may vary across cultures. It is thus important to test whether the hedonic and the eudaimonic motives of Chinese people relate to the two kinds of well-being in a similar way as those of Western people.

The research regarding happiness motives that have been conducted in collectivist cultures is scanty, thus yielding inconclusive findings. Following Huta (2016) revised framework that differentiates between seeking pleasure and seeking comfort in hedonic motives, Asano et al. (2018) found that, among Japanese people, enhanced pleasure motives and eudaimonic motives predicted an increased positive affect and meaning in life after 2 months, while enhanced comfort motives did not predict any well-being outcomes; however, this study examined different types of motives separately, and little is known about their relative effects. The study of Lai et al. (2020), although assessing happiness motives, examined the moderation role of happiness motives in the effects of prosocial behavior on SWB by manipulating prosocial behavior with an experimental design. Thus, it could not inform the direct association between happiness motives and SWB.

Research based on the framework of orientations to happiness provides implications for understanding the relationship between happiness motives and well-being in Chinese context. It is found that eudaimonic orientations (i.e., life of meaning) demonstrate stronger associations with well-being than hedonic orientations (i.e., life of pleasure) (Chen, 2010; Chan, 2013; Yang et al., 2017). Taken together, we predicted that both hedonic and eudaimonic motives would contribute to well-being, but eudaimonic motives would make a greater contribution than hedonic motives in an Eastern collectivist culture.

### Mediation of Satisfaction and Frustration of Basic Psychological Needs

Another lacuna in the literature of happiness motives is the mechanisms underlying the associations between these motives and well-being. According to BPNT (Ryan and Deci, 2000), the crux of SDT (Ryan and Deci, 2017), the satisfaction and the frustration of basic psychological needs may link happiness motives (i.e., means) to SWB/EWB (i.e., ends). BPNT posits that human beings are subject to the three basic psychological needs of autonomy, relatedness, and competence. Autonomy involves the need to act according to one’s authentic self rather than external forces. Relatedness reflects the need to have intimate and genuine connections with others and to belong to a group. Competence concerns the need to feel efficacious and capable of achieving the desired goals (Ryan and Deci, 2017).

People experience positive emotions, obtain “nutriments” for further growth, and become fully functional when these needs are satisfied (Ryan and Deci, 2000). Supporting this argument, numerous studies have found positive associations between need satisfaction and SWB/EWB in both general and specific domains, including education, work, sports, and health [for reviews, see Ng et al. (2012), Van den Broeck et al. (2016), and Martela and Sheldon (2019)]. Such positive links are generalizable to both individualist and collectivist cultures (Church et al., 2013; Chen et al., 2015; Yu et al., 2018).

In addition, differently from lacking need satisfaction, recent research has proposed need frustration as an asymmetrical dimension of need experience that indicates a more active obstruction of needs (Vansteenkiste and Ryan, 2013). For example, people whose needs for relatedness could not be satisfied do not have a strong connection with significant others, while people whose need for relatedness is frustrated experience hostility or indifference from their significant others. People who feel that their basic needs are deprived or blocked become vulnerable to risk factors and deficient in the psychological resources to cope with adversity; thus, they are more likely to show defensiveness and pathological functioning and less likely to experience happiness (Chen et al., 2015; Costa et al., 2016; Longo et al., 2016). Research repeatedly supported the distinction between need satisfaction and need frustration and found that need frustration has a unique association with ill-being (e.g., depression, exhaustion, and negative affect; Longo et al., 2016, 2018; Tindall and Curtis, 2019) relative to need satisfaction. The study of Martela and Ryan (2020) also showed that need frustration negatively related to EWB. The unique role of need frustration has been also supported in different cultural contexts (e.g., Unanue et al., 2014; Chen et al., 2015). For example, Chen et al. (2015) found that need satisfaction was related to more life satisfaction and vitality and fewer depressive symptoms, while need frustration was related to less life satisfaction and more depressive symptoms, and this was found to be true for people across four nations (e.g., United States, Belgium, China, and Peru).

Given the roles that basic needs play in reflecting the extent to which individuals experience satisfaction or frustration from their activities and environments, need satisfaction and need frustration presumably serve as a bridge connecting ways of living and well-being (Martela and Sheldon, 2019). Happiness motives are translated into feelings or experiences of happiness because they drive people to engage in activities that help fulfill the basic needs for autonomy, relatedness, and competence. Pursuing hedonia represents self-care that enables individuals to rest and return with a fresher perspective, while pursuing eudaimonia represents self-development that enables individuals to act on their values and actualize their goals (Huta, 2016). Accordingly, hedonic motives drive individuals to engage in activities that allow them to take care of their basic needs for autonomy, relatedness, and competence, while eudaimonic motives drive individuals to engage in activities that further develop their autonomy, relatedness, and competence.

It is reasonable to expect that eudaimonic motives would contribute to well-being through need experience. As argued by Deci and Ryan (2013), “eudaimonic living fosters well-being because it provides satisfaction of people’s most fundamental needs” (p. 135). People who pursue eudaimonia tend to engage in activities that tap into their deep psychological needs, which further contributes to their well-being. In line with this view, studies showed that eudaimonic motives were positively related to the satisfaction of each of the three basic needs [see Huta (2016)]. One study found that pursuing intrinsic life goals (e.g., belonging and personal growth)—a eudaimonic way of living—was related to increased need satisfaction relative to extrinsic life goals (e.g., fame and wealth) (Hope et al., 2019). Given the robust evidence showing the positive link between need satisfaction and well-being, the mediating role of need satisfaction should be established.

Comparatively, the relationship between hedonic motives and need satisfaction might be debatable. On the one hand, hedonic living possibly brings individuals momentary positive emotions directly without influencing their need experience. On the other hand, it may contribute to a more stable and positive cognitive appraisal of life as it takes care of basic psychological needs. For instance, people who seek for relaxation actually enjoy a period of time focusing on self-interest and being free of external force, which fulfils the need for autonomy. Supporting this view, studies showed that hedonic motives were positively related to the satisfaction of each of the three basic needs [see Huta (2016)]. Another study showed that focusing on present hedonism (i.e., a time perspective that focuses on immediate pleasure) was positively related to need satisfaction (Zhang et al., 2013). Therefore, this research would explore these two possibilities by empirically testing the direct effect and the indirect effect of hedonic motives on SWB.

Furthermore, happiness motives develop into well-being because they drive people to engage in activities that prevent them from frustrating their basic needs. The relationship between happiness motives and need frustration is not yet fully known. Nevertheless, given the independent role of need frustration in human functioning, it is necessary to consider it in the mechanisms that relate happiness motives to well-being outcomes. It is reasonable to believe that people with strong hedonic motives avoid participating in activities that potentially threaten their basic needs. People who often seek relaxation also tend to avoid engaging in competition that may threaten their competence. Furthermore, eudaimonic motives can guide people to activities that will help them accumulate psychological resources, such as social bonding or the development of sophisticated skills for coping with situations or environments that thwart their needs. Therefore, both hedonic and eudaimonic motives may negatively relate to need frustration. Taken together, we contended that both hedonic and eudaimonic motives may positively relate to SWB/EWB *via* heightened need satisfaction and lessened need frustration. In other words, both SWB and EWB can serve as a gauge of the extent to which ways of living fulfill or frustrate basic needs. However, no studies have empirically tested the role of need experience in the relationship between ways of living and well-being. Thus, we explored these indirect effects in the current research.

### Current Research

The studies of the current research aimed at filling two gaps in the literature on well-being: (1) Which happiness motives (hedonic vs. eudaimonic) are more conducive to SWB and EWB among Chinese people? (2) How do different types of motives contribute to SWB and EWB? SWB was indexed by life satisfaction, an overall cognitive appraisal of life conditions (Diener et al., 1999), and EWB was indexed by meaning in life, the subjective experience that “life is meaningful” (Heintzelman and King, 2014). We clearly differentiated between hedonia and eudaimonia for both motives and outcomes. These study outcomes are essential to the concept of SWB and EWB, respectively (Diener et al., 1999; Steger et al., 2006), and reflect subjective feelings and experiences of well-being without being mixed up with ways of living. In study 1, we attempted to address the first question by testing the following hypotheses:

(H1) Hedonic motives will be positively associated with life satisfaction (hypothesis 1).
(H2) Eudaimonic motives will be positively associated with both life satisfaction and meaning in life (hypotheses 2a and 2b).
(H3) Eudaimonic motives will have stronger relationships with life satisfaction and meaning in life relative to hedonic motives (hypotheses 3a and 3b).

Then, in study 2, we attempted to address the second question by testing the following hypotheses:

(H4) Hedonic motives will have an indirect effect on life satisfaction *via* need satisfaction and need frustration, such that hedonic motives will positively relate to life satisfaction through more need satisfaction (hypotheses 4a) and less need frustration (hypotheses 4b).
(H5) Eudaimonic motives will have indirect effects on life satisfaction/meaning in life *via* need frustration and need satisfaction, such that eudaimonic motives will positively relate to life satisfaction/meaning in life through more need satisfaction (hypotheses 5a) and less need frustration (hypotheses 5b).

## Study 1

Study 1 aimed at understanding the associations between happiness motives and well-being, especially in regard to the relative effects of hedonic and eudaimonic motives on SWB/EWB in a Chinese context.

### Methods

#### Participants and Procedure

We recruited the participants *via* the Sojump platform^1^, which is similar to Amazon’s Mechanical Turk (Buhrmester et al., 2011) and provides an all-in-one solution for participant recruitment for online surveys in China. A total of 310 Chinese university students participated in this study. However, nine participants did not pass one of the two attention check questions^2^ [see Oppenheimer et al. (2009)], which resulted in 301 valid cases (226 females; mean age = 20.32 ± 1.60). The participants were requested to place themselves on a socioeconomic ladder with 10 rungs to indicate their relative socioeconomic status (SES) in Chinese society using the MacArthur Scale of Subjective Social Status (Adler et al., 2000). The average subjective SES was 4.56 (*SD* = 1.38). The ethical approval of this study had been obtained from the Human Subjects Ethics Sub-committee of University Research Committee in the corresponding author’s affiliated institution. All the participants provided their informed consent before participating in the study.

#### Instruments

##### Happiness motives

The participants reported their hedonic and eudaimonic motives *via* the revised Hedonic and Eudaimonic Motives for Activities Scale (Huta, 2016). Following the recommended translation and back-translation procedures (Brislin, 1980), we generated a Chinese version of this scale. The instructions were modified slightly to fit the Chinese language. The participants were asked to rate how often (rather than how much) they have various intentions when they approach daily activities using a seven-point scale (1 = never, 7 = always). Six items were representative of hedonic motives (e.g., “seeking enjoyment,” and “seeking relaxation”), and five items were representative of eudaimonic motives (e.g., “seeking to develop a skill, learn, or gain insight into something,” “seeking to contribute to others or the surrounding world”). We performed an exploratory factor analysis with principal axis factoring as the extraction method and Promax with Kaiser normalization as the rotation method to validate the structural validity of the scale. Both the scree plot and the eigenvalues suggested a two-factor solution. The first factor (λ = 3.21) reflected hedonic motives, explaining 29.14% of the variance. The factor loadings ranged from 0.56 to 0.75. The second factor (λ = 2.17) reflected eudaimonic motives, explaining 19.69% of the variance. The factor loadings ranged from 0.55 to 0.67. The internal consistency of this scale was good (see Table 2). The online Appendix 1 shows the factor loading for each item.

**TABLE 2:** Descriptive information and correlations of study variables in study 1 (*N* = 301).

	Mean (*SD*)	α	1	2	3	4	5	6
Hedonic motives	5.10 (0.98)	0.86	−					
Eudaimonic motives	4.68 (1.04)	0.79	–0.10	−				
Life satisfaction	3.43 (1.03)	0.85	0.02	0.42***	−			
Presence of meaning	4.94 (1.20)	0.89	–0.07	0.43***	0.51***	−		
Age	20.32 (1.60)	–	–0.07	0.12*	–0.02	–0.003	−	
Gender	–	–	–0.10	0.06	0.05	0.15*	−0.08	–
Subjective SES	4.56 (1.38)	–	–0.001	0.31***	0.38***	0.30***	−0.03	0.02

*Gender: 1, male; 0, female; SES, socioeconomic status.* *p < 0.05, ***p < 0.001.

##### Well-being

The participants reported their overall evaluation of life satisfaction *via* the Chinese version of the Satisfaction With Life Scale (Diener et al., 1985) as translated and validated by Shek (2004). The participants indicated the degree to which each statement was true of them (e.g., “The conditions of my life are excellent.”) using a six-point Likert scale (1 = not true at all; 6 = totally true). In addition, the participants reported their meaning in life using the subscale for the presence of meaning in the Meaning in Life Questionnaire (MLQ; Steger et al., 2006) (e.g., “My life has a clear sense of purpose.”) using a seven-point Likert scale (1 = not true at all; 7 = totally true). The MLQ has been widely used in various studies across different populations, including Chinese people (e.g., Wang et al., 2016). To validate the differentiation of life satisfaction and meaning in life, we performed a two-factor confirmatory factor analysis (CFA) *via* R programming (Lavaan; Rosseel, 2011). We evaluated the model fit using three commonly used fit indexes: the comparative fit index (CFI; acceptable fit: ≥0.90; good fit ≥0.95), Tucker–Lewis index (TLI; acceptable fit: ≥0.90; good fit ≥0.95; Hu and Bentler, 1999), and root-mean-square error of approximation (RMSEA; reasonable fit: 0.05–0.08; close fit ≤0.05; Browne and Cudeck, 1993). The model fitted the data well [CFI = 0.99, TLI = 0.98, RMSEA = 0.05, χ^2^(34) = 54.38]. Additionally, when the covariance of life satisfaction and meaning in life was constrained to be 1, the model fit significantly dropped [Δχ^2^(1) = 23.77,*p* < 0.001], which indicated that life satisfaction and meaning in life should be two distinct constructs. The internal consistencies of the two measures were good (see Table 2).

### Results and Brief Discussion

**FIGURE 1:**
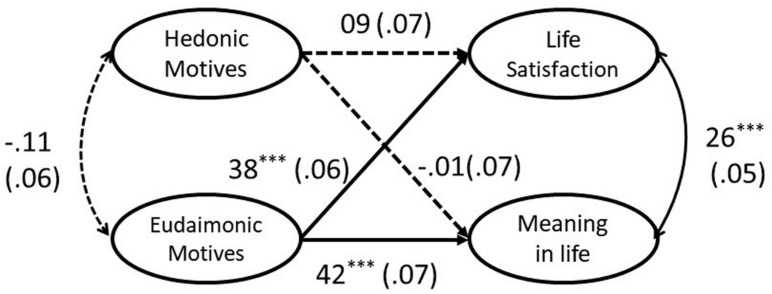
Model of the relationship between happiness motives and well-being. This figure presents the relationships between happiness motives and well-being outcomes and the results. The ellipses represent latent factors. In the SEM analysis, each latent factor was indexed by the items of the corresponding scale. The participants’ gender, age, and subjective socioeconomic status were allowed to predict life satisfaction and meaning in life while omitted in the figure. The numbers represent unstandardized estimated coefficients and their standard errors (in brackets). The dotted lines mean non-significant coefficients. ***p < 0.001.

As shown in Table 2, hedonic motives were associated with neither life satisfaction nor meaning in life, while eudaimonic motives were positively associated with both. These two types of motives were not significantly related to each other. To understand their relative associations with well-being, structural equation modeling (SEM) was used to examine the relative effects of hedonic and eudaimonic motives on both indicators of well-being (see Figure 1). The model contains four latent factors of hedonic motives, eudaimonic motives, life satisfaction, and meaning in life. The model yielded acceptable fit [CFI = 0.92, TLI = 0.91, RMSEA = 0.06, χ^2^(239) = 483.84]. Supporting hypotheses 2 and 3, the results showed that, after controlling age, gender, and subjective SES, the eudaimonic motives were positively associated with life satisfaction (β = 0.41) and meaning in life (β = 0.43), whereas the hedonic motives were not related to life satisfaction and meaning in life. Our findings did not support hypothesis 1.

Consistent with previous studies (Huta, 2016), eudaimonic motives seem to convey benefits for both SWB and EWB. Unexpectedly, hedonic motives made little contribution to well-being. These results echo other studies using Chinese adolescent or adult samples (Chan, 2013; Yang et al., 2017), indicating that, relative to a hedonic orientation (i.e., life of pleasure), a eudaimonic orientation (i.e., life of meaning) to happiness demonstrated stronger associations with subjective well-being (i.e., life satisfaction, positive affect, and negative affect). However, the participants were mostly female students. We thus tested whether these findings could be replicated in another sample. Furthermore, study 1 did not explain how eudaimonic motives were associated with well-being. Therefore, study 2 attempted to extend our understanding regarding the mechanisms underlying these associations.

## Study 2

The aims of study 2 were twofold. The first aim was to replicate the results of study 1 and the second was to investigate the mediating role of psychological need satisfaction and frustration in the link between happiness motives and well-being.

### Methods

#### Participants and Procedure

*Via* the Sojump platform, we recruited 527 college students who did not participate in study 1. All the participants passed the two attention check questions; however, one was of age 33, which was out of the typical age range for college students and was thus removed from the analyses. This resulted in a sample of 526 college students (275 females; mean age = 20.19 ± 1.57). The average subjective SES of this sample was 4.44 (*SD* = 1.42). Ethical approval of this study had been obtained from the Human Subjects Ethics Sub-committee of the University Research Committee in the corresponding author’s affiliated institution. All the participants provided their informed consent before participating in the study.

#### Instruments

##### Happiness motives

We used the same measures as in study 1. As Huta (2016) proposed a three-factor model by further differentiating two types of hedonic motives—pleasure motives and comfort motives, we performed a CFA to validate the structural validity of the scale. We compared two nested models with three latent factors of eudaimonic motives, pleasure motives, and comfort motives. The first model allowed for the free estimation of correlations among the three latent factors, while the second model constrained the covariance between pleasure motives and comfort motives to be 1, representing a two-factor model. The first model fitted the data well [CFI = 0.98, TLI = 0.97, RMSEA = 0.05, χ^2^(41) = 94.87] and showed a high correlation between pleasure motives and comfort motives (*r* = 0.83, *p* < 0.001). Nonetheless, the χ^2^ change test showed that the constraint did not result in a significant reduction of the model fit [Δχ^2^(1) = 0.04, *p* > 0.05], which indicated that a two-factor model [CFI = 0.98, TLI = 0.97, RMSEA = 0.05, χ^2^(42) = 94.91] fitted the data as well as the three-factor model. For parsimony, we adopted the two-factor solution as in study 1. We re-run a two-factor model that consists of two latent factors of hedonic and eudaimonic motives and presented the factor loadings in the online Appendix 1. The internal consistency of this scale was satisfactory (see Table 3).

**TABLE 3:** Descriptive information and correlations of study variables in study 2 (*N* = 526).

	Mean (SD)	α	1	2	3	4	5	6	7	8	9	10	11	12
Hedonic motives	4.72 (1.09)	0.87	−											
Eudaimonic motives	5.10 (0.94)	0.77	−0.15***	−										
Life satisfaction	3.29 (0.92)	0.81	−0.11*	0.32***	−									
Presence of meaning	4.75 (1.19)	0.89	−0.15***	0.44***	0.47***	−								
Autonomy satisfaction	4.22 (0.80)	0.73	–0.04	0.47***	0.48***	0.46***	−							
Relatedness satisfaction	4.75 (0.88)	0.78	–0.08	0.34***	0.43***	0.37***	0.42***	−						
Competence satisfaction	4.47 (0.82)	0.81	−0.14**	0.49***	0.42***	0.50***	0.51***	0.49***	−					
Autonomy frustration	3.24 (1.01)	0.81	0.24***	−0.32***	−0.41***	−0.36***	−0.53***	−0.37***	−0.42***	−				
Relatedness frustration	2.49 (0.89)	0.74	0.04	−0.21***	−0.28***	−0.26***	−0.29***	−0.51***	−0.35***	0.40***	−			
Competence frustration	3.38 (1.12)	0.84	0.20***	−0.36***	−0.47***	−0.46***	−0.46***	−0.38***	−0.62***	0.52***	0.44***	−		
Age	20.19 (1.57)	−	−0.09*	–0.002	0.06	0.06	0.00	0.03	0.06	–0.04	−0.15***	−0.11*	−	
Gender	1.52 (0.50)	−	0.04	0.03	0.11*	0.13**	0.08	–0.07	0.09*	0.01	–0.02	−0.14**	0.04	−
Subjective SES	4.44 (1.42)	−	−0.11*	0.24***	0.34***	0.21***	0.27***	0.27**	0.25***	−0.21***	−0.16***	−0.30***	0.07	0.001

Gender: 1, male; 0, female; SES, socioeconomic status. *p < 0.05, **p < 0.01, ***p < 0.001.

##### Well-being

We used the same measures for life satisfaction and meaning in life as in study 1. Their internal consistencies were good (see Table 3). Similar to the findings of CFA in study 1, life satisfaction and meaning in life were found to be two distinct factors [CFA = 0.98, TLI = 0.97, RMSEA = 0.05, χ^2^(34) = 82.01; Δχ^2^(1) = 45.92, *p* < 0.001].

##### Psychological need satisfaction and frustration

We used the Basic Psychological Need Satisfaction and Frustration Scale. Chen et al. (2015) created and validated this scale across cultures, including the Chinese culture. The participants reported their satisfaction of needs for autonomy (“I feel a sense of choice and freedom in the things I undertake.”), relatedness (e.g., “I feel that the people I care about also care about me.”), and competence (“I feel capable at what I do.”) and their frustration of needs for autonomy (“I feel pressured to do too many things.”), relatedness (“I feel excluded from the group I want to belong to.”), and competence (e.g., “I feel insecure about my abilities.”) using a six-point scale (1 = not true at all; 6 = totally true). A higher-order CFA analysis was performed for the scale. Autonomy satisfaction, relatedness satisfaction, competence satisfaction, autonomy frustration, relatedness frustration, and competence frustration represented the first-order latent factors. Each first-order latent factor was indexed by the scores of individual items. Need satisfaction and need frustration represented two second-order latent factors, with the former indexed by three first-order factors of need satisfaction and the latter indexed by three first-order factors of need frustration. This model yielded an acceptable fit [CFA = 0.92, TLI = 0.91, RMSEA = 0.06, χ^2^(249) = 659.29], which evidenced the structural validity of the scale. Additionally, when the covariance of the second-order factors was constrained to be 1, the constrained model demonstrated a significantly worse model fit [Δχ^2^(1) = 1,015.2, *p* < 0.001], which indicated that need satisfaction and need frustration were two distinct factors. The internal consistency of each sub-scale was good (see Table 3).

### Results and Brief Discussion

#### Simple Correlations

Table 3 shows the correlations among the study variables. Consistent with study 1, eudaimonic motives were positively associated with meaning in life and life satisfaction. However, unlike in study 1, hedonic motives were negatively related to life satisfaction and meaning in life. Additionally, bivariate correlation analyses showed that hedonic motives were negatively related to competence satisfaction while positively related to autonomy frustration and competence frustration. As expected, eudaimonic motives were positively related to the satisfaction of all three needs and negatively related to the frustration of all three needs. In addition, need satisfaction was positively associated while need frustration was negatively associated with life satisfaction and meaning in life.

#### The Mediating Roles of Psychological Need Satisfaction and Frustration

**FIGURE 2:**
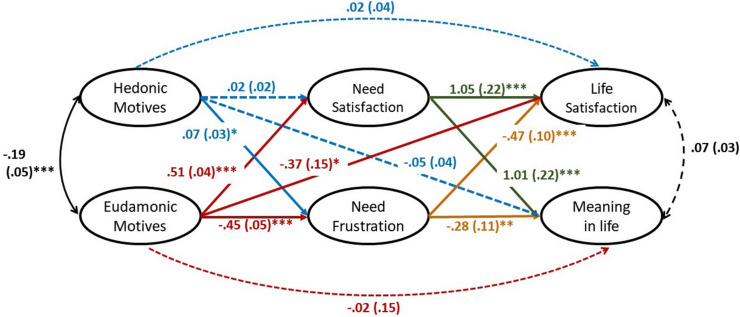
Model of the relationship between happiness motives and well-being *via* need experience. This figure presents a mediation model with happiness motives as independent variables, need experience as mediators, and well-being as dependent variables and the results. The ellipses represent latent factors. In the SEM analysis, need satisfaction was indexed by autonomy satisfaction, relatedness satisfaction, and competence satisfaction, while need frustration was indexed by autonomy frustration, relatedness frustration, and competence frustration. All other latent factors were indexed by the items of the corresponding scale. The participants’ gender, age, and subjective socioeconomic status were allowed to predict need satisfaction, need frustration, life satisfaction, and meaning in life while omitted in the figure. The numbers represent unstandardized estimated coefficients and their standard errors (in brackets). The dotted lines mean non-significant coefficients. *p < 0.05, **p < 0.01, ***p < 0.001.

We performed SEM analysis *via* R programming (Lavaan; Rosseel, 2011) to examine the mediating roles of need satisfaction and need frustration simultaneously. Figure 2 shows the SEM model and the results of the structural path coefficients. Both the direct effects and the indirect effects of hedonic motives and eudaimonic motives on life satisfaction and meaning in life were estimated. We controlled the participants’ age, gender, and subjective SES in the model. We used bias-correlated bootstrapping with 10,000 samples to estimate the 95% confidence interval (CI) of indirect effect. A CI excluding zero indicates a significant indirect effect (Hayes, 2013).

The model showed acceptable fit [CFA = 0.92, TLI = 0.91, RMSEA = 0.05, χ^2^(432) = 6,985.48]. Regarding hedonic motives, there was only one significant indirect effect. There were no significant indirect effects through need satisfaction. The indirect effect of hedonic motives on meaning in life through need frustration was not significant. However, the indirect effect of hedonic motives on life satisfaction through need frustration was significant [*b* = −0.03, *SE* = 0.02, β = −0.04, *p* = 0.04, 95%CI = (−0.07, −0.002)]. Hedonic motives were positively associated with need frustration (β = 0.11), which was in turn negatively associated with life satisfaction (β = −0.37). There were no significant direct effects of hedonic motives on life satisfaction and meaning in life. Additionally, the indirect effects of eudaimonic motives on life satisfaction [*b* = 0.53, *SE* = 0.12, β = 0.55, *p* < 0.001, 95%CI = (0.30, 0.77)] and meaning in life [*b* = 0.51, *SE* = 0.12, β = 0.45, *p* < 0.001, 95%CI = (0.28, 0.75)] through need satisfaction were both significant. Eudaimonic motives were positively related to need satisfaction (β = 0.81), which was in turn positively related to life satisfaction (β = 0.68) and meaning in life (β = 0.56), respectively. Besides that, the indirect effects of eudaimonic motives on life satisfaction [*b* = 0.21, *SE* = 0.05, β = 0.22, *p* < 0.001, 95%CI = (0.11, 0.31)] and meaning in life [*b* = 0.13, *SE* = 0.05, *p* < 0.01, β = 0.11, 95%CI = (0.03, 0.22)] through need frustration were both significant. Eudaimonic motives were negatively related to need frustration (β = −0.59), which was in turn negatively related to life satisfaction (β = −0.37) and meaning in life (β = −0.14), respectively. The direct effect of eudaimonic motives on meaning in life was not significant, but that on life satisfaction was negative and significant. The negative direct effect might indicate a potential process in which eudaimonic motives do harm to life satisfaction but not *via* the mediation of need experience. For example, if people hold eudaimonic motives to an extreme that they always scarify the gratification of their immediate enjoyment to achieve excellence in the future, they might not feel good about their current lives. Identifying such a negative pathway is possibly useful for developing a healthy way to achieve eudaimonic motives. Thus, we call for scholars’ attention to the potential dark side of eudaimonic motives and await future studies to replicate the current findings and investigate the possible mechanisms. Accordingly, hypotheses 5 (but not hypotheses 4) were supported. Together with prior research (e.g., Boone et al., 2014; Wang et al., 2017), this study has demonstrated that not only need satisfaction but also need frustration can explain the relationship between how people live their lives and how they experience happiness.

## General Discussion

Throughout history, there has been an ongoing and lively debate regarding what makes life worthwhile. To answer this question, we need not only to study a good life as an outcome but also to investigate what should be done to achieve a good life. With the refinement of the conceptualization of happiness or well-being, researchers have differentiated between eudaimonia and hedonia (Ryan and Deci, 2001) and between way of living and outcomes (Huta and Waterman, 2014). What follows is a critical question about which way of living facilitates the attainment of which type of happiness outcome. Based on two samples of Chinese college students, our studies found that people who hold stronger eudaimonic motives in daily activities experienced better SWB and EWB, while those who hold stronger hedonic motives did not show better well-being. More importantly, we found that the satisfaction and the frustration of needs for autonomy, relatedness, and competence could explain these associations.

The current findings lend support to the distinction between hedonia and eudaimonia in motives for actions. The dichotomization of well-being into hedonic and eudaimonic perspectives is well-documented yet debatable in positive psychology (Keyes and Annas, 2009; Huta and Waterman, 2014). Despite that numerous studies have demonstrated their differences in conceptualization and psychosocial correlates (e.g., Waterman et al., 2008; Delle Fave et al., 2011), some scholars tend to support an integrated concept of well-being (e.g., Keyes and Annas, 2009; Diener et al., 2010; Disabato et al., 2016). Our research yields supportive evidence for a hedonia–eudaimonia distinction in happiness motives. Relative to hedonic motives, we consistently found that eudaimonic motives were more conducive to well-being, regardless of SWB or EWB. In line with previous studies (e.g., Yang et al., 2017; Kryza-Lacombe et al., 2019), our study suggests that living in a eudaimonic manner is an important approach for creating a satisfying and meaningful life. In contrast, hedonic motives appeared not beneficial to well-being. Even though some researchers prefer using an integrated concept (e.g., Flourishing; Keyes and Annas, 2009) to evaluate people’s overall mental well-being, it is still important to differentiate the motives behind the actions.

The unexpected findings of hedonic motives also suggest potential cultural variations in the outcomes of hedonic ways of living. As the first study to investigate the associations between happiness motives and well-being in Chinese context, it unfolds both the similarities and the differences in associations between happiness motives and well-being as compared with the research findings of previous studies predominantly using Western samples. We observed positive associations of eudaimonic motives and life satisfaction/meaning in life. Similar to the case in Japan (Asano et al., 2018), the associations can be regarded as large in the literature of social and personality psychology (Funder and Ozer, 2019). The association between eudaimonic motives and life satisfaction (*r*s = 0.32–0.42) appeared larger than that of the four studies (*r*s = 0.09–0.26) of Huta and Ryan (2010) based on American samples, while the association between eudaimonic motives and meaning in life (*r*s = 0.42–0.44) fell in the range of those found in their studies (*r*s = 0.19–0.58). The benefits of eudaimonic motives are typically true in East Asian culture, where people tend to consider interpersonal harmony (Lu and Gilmour, 2004; Uchida and Kitayama, 2009) and self-cultivation (Joshanloo, 2014) as keys to achieving a happy and virtuous life. Therefore, Chinese people may find it more appropriate and be more encouraged by their culture to pursue happiness in a eudaimonic manner than in a hedonic one. However, the associations between hedonic motives and well-being were out of our expectation and incongruent with prior studies (e.g., Chen, 2010; Asano et al., 2018). Hedonic motives made little contribution to well-being in our studies. Study 2 even showed negative correlations between hedonic motives and well-being indicators, though the effect size was small. Hedonic motives may not be socially desirable in a Chinese society. Supporting this view, our unpublished study using a group of Chinese college students (*N* = 319) showed that social desirability was negatively associated with hedonic motives (*r* = −0.14, *p* < 0.05) but positively associated with eudaimonic motives (*r* = 0.19, *p* < 0.001). Due to this conflict with cultural values, people who often use hedonic motives in daily life may experience internal (e.g., guilt) or external pressures (e.g., negative social evaluation). Furthermore, they may also encounter social constraints that hinder them from actualizing their hedonic intentions even if they hold such behavioral intentions. Much work has been done to address the question about what accounts for a happy life across cultures (e.g., Church et al., 2014). Nevertheless, this research further indicates a need to use a cross-cultural perspective to understand how people pursue happiness. It is noted that the current research is not based on a direct cultural comparison. Given the mixed findings in our studies, we encourage researchers to further examine the moderating role of cultural values on the link between hedonic motives and well-being and to investigate whether the attainment of hedonic motives plays a role in this relationship.

More importantly, we have taken an initial step toward explicating the mechanisms underlying the relationship between happiness motives and well-being. The use of HEMA framework in this research advances our understanding of the pathway to well-being. Previous research that investigated orientations to happiness has provided us a rich profile (e.g., what do they think? what do they behave? how do they feel about their lives?) of individuals who live in hedonic and eudaimonic ways, respectively (e.g., Peterson et al., 2005). By focusing on happiness motives, the current findings indicate a possible process about how individuals pursue and achieve well-being step by step. Specifically, we found that need satisfaction linked eudaimonic motives to both SWB and EWB. These findings were in line with previous studies that examined how need satisfaction intervenes in the relationship between a non-eudaimonic way of living and well-being. For example, a three-wave longitudinal study showed that materialism (i.e., considering material possessions as central to happiness and success) predicted decreased life satisfaction and increased depression *via* decreased need satisfaction (Wang et al., 2017). People who hold stronger eudaimonic motives tend to engage in activities that express personal values and beliefs, contribute to the welfare of others, or lead to the acquisition of new knowledge and skills. These activities presumably help satisfy the needs for autonomy, relatedness, and competence; thus, they enhance both SWB and EWB.

Furthermore, by simultaneously examining need satisfaction and need frustration, we showed that need frustration plays a unique role above and beyond a lack of need satisfaction. Specifically, eudaimonic motives related to SWB/EWB and hedonic motives related to SWB *via* need frustration. Going beyond previous studies showing that lessened need satisfaction and heightened need frustration were both harmful to well-being (e.g., Chen et al., 2015; Costa et al., 2016), this research suggests that these two aspects of need experience serve as two distinct bridges that connect ways of living and well-being outcomes. These two need experiences serve different mechanisms. A lack of need satisfaction fails to energize individuals for growth (i.e., strength-oriented), whereas need frustration enhances the vulnerability of individuals to illness and psychopathology (i.e., deficit-oriented) (Vansteenkiste and Ryan, 2013). Happiness motives that benefit well-being may either enhance the potential for growth or reduce the risks for malfunctioning. It is interesting to find that both need satisfaction and need frustration explained the link between eudaimonic motives and SWB/EWB, but only need frustration explained the link between hedonic motives and SWB. Further studies are needed to retest if hedonic motives convey more effects on need frustration than need satisfaction. Moreover, it is necessary to examine how a hedonic way of living increases or reduces individuals’ experiences of relational exclusion, feelings of failure, and external and self-imposed pressure.

### Limitations and Future Directions

First, the cross-sectional design with self-report used by these two studies hinders us from drawing causal inference. The directionality of the links is thus unclear. Our research defined happiness motives as dispositional traits and measured them in a retrospective manner, and it thus is conceptually reasonable to hypothesize happiness motives as predictors of well-being. Nevertheless, it is still possible that people may adjust their happiness motives based on their chronic need experiences (Olafsen et al., 2018). Additionally, the associations among happiness motives, need experience, and well-being may be inflated due to common method variance. Therefore, future studies will benefit from an experimental design or a longitudinal design to validate the causal mechanisms and from the use of objective indicators of well-being to minimize the common method variance.

Second, our studies relied on college student samples, and therefore we should be cautious about generalizing from these results to other population groups. The study of McMahan and Estes (2011) on lay conceptions of well-being found that the hedonic conception of well-being (i.e., presence of pleasure and avoidance of negative experience) had a stronger effect on well-being among older adults relative to younger adults, but the effects of a eudaimonic conception of well-being (i.e., self-development and contribution to others) seemed to be age-invariant. Accordingly, it is likely that the relationship between hedonic motives and well-being will change with age. It is necessary to consider individual differences in future studies.

Third, meaning in life was used to indicate eudaimonic well-being in the current study, but it is not the single indicator of eudaimonic well-being (Ryff and Keyes, 1995; Diener et al., 2010). Outcomes of eudaimonic well-being include positive experiences (e.g., feeling of personal expressiveness, Waterman et al., 2008) and functioning (e.g., environmental mastery; Ryff and Keyes, 1995; Huta and Waterman, 2014). To extend the current findings, future studies need to measure other experiences of eudaimonic well-being. Additionally, the current study did not include ill-being or psychopathology as an outcome. It is unclear whether hedonic motives will enhance the likelihood of ill-being and eudaimonic motives will reduce it. Previous research has found that need frustration is more related to ill-being than need satisfaction (e.g., Longo et al., 2016). It is possible that need frustration is a stronger mediator that relates happiness motives to the negative side of mental health. Therefore, future research would benefit from an inquiry into ill-being outcomes.

Finally, without including different cultural samples, the implications for cultural variations yielded by our research are limited. We inferred the possible cultural comparison merely by referring to the previous studies using Western samples. Previous research has pointed out the possibility that hedonism is downplayed in Chinese culture (Lu and Gilmour, 2004; Joshanloo and Jarden, 2016), but the current studies did not test this possibility directly. Similar to our studies, the majority of the previous studies that examined the relationship between happiness motives and well-being relied on college student samples (see Table 1). However, it is unknown whether the differences found in our studies can be attributed to the socio-demographic background (e.g., SES) of the college student samples. Therefore, we encourage future studies to investigate different cultural samples simultaneously and identify the conditions on which hedonic motives benefit/impair one’s well-being.

## Conclusion

The current studies revealed that eudaimonic motives, not hedonic motives, contributed to SWB and EWB in Chinese subjects. Furthermore, need satisfaction and need frustration explained the relationship between eudaimonic motives and SWB and EWB, while need frustration explained the relationship between hedonic motives and SWB. These findings suggest potential cultural differences in the relationship between hedonic motives and well-being and highlight the important role of experience in the needs for autonomy, relatedness, and competence when translating happiness motives into well-being outcomes. We call for more studies to consider the role of cultural contexts in happiness pursuit and to unravel the process from happiness pursuit to happiness attainment.

## Data Availability Statement

The datasets generated for this study are available on request to the corresponding author.

## Ethics Statement

The studies involving human participants were reviewed and approved by Human Subjects Ethics Sub-committee of The Hong Kong Polytechnic University. The patients/participants provided their written informed consent to participate in this study.

## Author Contributions

LL designed the study, analyzed and interpreted the data, and drafted the manuscript. H-WC co-designed the study, participated in the interpretation of results, and edited the manuscript. Both authors contributed to the article and approved the submitted version.

## Conflict of Interest

The authors declare that the research was conducted in the absence of any commercial or financial relationships that could be construed as a potential conflict of interest.

## Footnotes

1 sojump.com

2 Instructional manipulation check is often used in online survey to assess the participants’ attentiveness to the instructions. In this study, the participants were requested to choose the option as instructed (e.g., “This is an attentional check, and please choose ‘not true at all’ for this item”).
