# Supplementary material for: The Associations Between Happiness Motives and Well-Being in China: The Mediating Role of Psychological Need Satisfaction and Frustration
Source: Front Psychol. 2020 Sep 3;11:2198. doi: 10.3389/fpsyg.2020.02198 (PMC7495498; doi:10.3389/fpsyg.2020.02198)
Supplement: Supplementary file 1 [file Table_1.DOCX]

Supplementary Material

Appendix 1. Results of Exploratory Factor Analysis (EFA) and Confirmatory Factor Analysis (CFA)

Table S1. Factor loadings of EFA

|  | Factor 1 | Factor 2 |
| --- | --- | --- |
| 1. Seeking to pursue excellence or a personal ideal |  | .685 |
| 1. Seeking enjoyment | .714 |  |
| 1. Seeking to use the best in yourself |  | .735 |
| 1. Seeking pleasure | .602 |  |
| 1. Seeking to develop a skill, learn, or gain insight into something |  | .694 |
| 1. Seeking fun | .713 |  |
| 1. Seeking to do what you believe in |  | .647 |
| 1. Seeking relaxation | .714 |  |
| 1. Seeking to contribute to others or the surrounding world |  | .576 |
| 1. Seeking to take it easy | .825 |  |
| 1. Seeking to have things comfortable | .708 |  |

Note. Extraction Method is Principal Axis Factoring. Rotation Method is Promax with Kaiser Normalization. Factor 1 reflects hedonic motives and Factor 2 reflects eudaimonic motives.

Table S2. Factor loadings of CFA for Hedonic and Eudaimonic Motives for Activities Scale

|  | Eudamonic | Hedonic |
| --- | --- | --- |
| 1. Seeking to pursue excellence or a personal ideal | .69 |  |
| 1. Seeking enjoyment |  | .73 |
| 1. Seeking to use the best in yourself | .73 |  |
| 1. Seeking pleasure |  | .56 |
| 1. Seeking to develop a skill, learn, or gain insight into something | .57 |  |
| 1. Seeking fun |  | .74 |
| 1. Seeking to do what you believe in | .69 |  |
| 1. Seeking relaxation |  | .79 |
| 1. Seeking to contribute to others or the surrounding world | .54 |  |
| 1. Seeking to take it easy |  | .80 |
| 1. Seeking to have things comfortable |  | .80 |

Note. The CFA analysis estimated the two-factor model. Estimation method is maximum likelihood. The latent factors are standardized estimates.
